# TILLCANN: a TILLING platform in *Cannabis sativa* for mutation discovery and crop improvement

**DOI:** 10.1186/s43897-025-00176-w

**Published:** 2025-10-13

**Authors:** Diana Duarte-Delgado, Konstantinos G. Alexiou, Marta Pujol, Cristobal Uauy, Nikolai M. Adamski, Victoria Vidal, Anthony Torres, Christopher Zalewski, Reginald Gaudino, Amparo Monfort, Jason Argyris

**Affiliations:** 1 Centre for Research in Agricultural Genomics (CRAG), CSIC-IRTA-UAB-UB, Campus UAB, Bellaterra, Barcelona, Spain; 2 IRTA (Institut de Recerca I Tecnologia Agroalimentàries), Barcelona, Spain; 3 John Innes Centre, Norwich Research Park, Norwich, NR4 7UH UK; 4 Front Range Biosciences, Lafayette, CO USA; 5 CIAT (International Center for Tropical Agriculture), Present address: Bean Program at Alliance of Bioversity International, Cali, Colombia; 6 Present address: Terpene Belt Farms, Oakland, CA USA; 7 Present Address: Cannabis Research Institute, Discovery Partners Institute, Chicago, IL USA

*Cannabis sativa* L is an emblematic multi-purpose crop species as a source of fibers from stalks, oil from seeds, and phytochemicals for medicinal and psychoactive purposes mainly produced in the female flowers (Andre et al., 2016). Historically, stringent regulations and selective breeding focused on a few specialized traits have contributed to reduce genetic variation and limit germplasm availability, while hindering the development of new genetic resources. TILLING (Targeting Induced Local Lesions in Genomes) is a commonly used strategy for creating novel artificial variation or diversification of traits for plant breeding (Tadele, 2016). However, there is no standardized and systematic protocol published for performing mutagenesis on *C. sativa*, and high-throughput mutation detection approaches have not previously been applied in the crop. We developed and optimized a standard EMS mutagenesis protocol to create a publicly available TILLING platform (TILLCANN) in *C. sativa* composed of 1,633 M2 families and employed TILLING by sequencing (TbyS) (Tsai et al., 2011) to rapidly discover mutations in agronomically important genes of interest (Fig. 1a).

**Fig.1:**
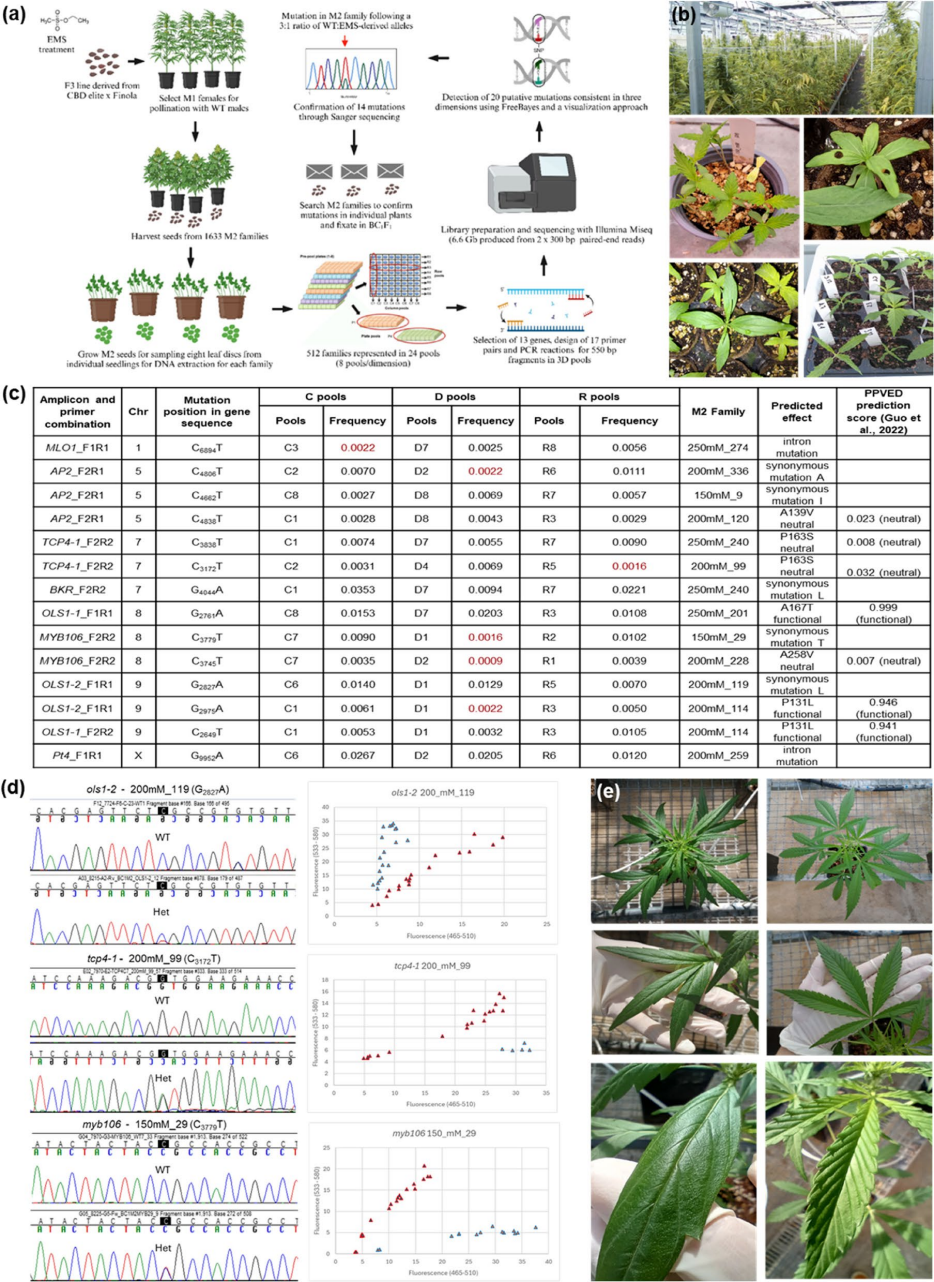
TILLCANN: a TILLING Platform in *Cannabis sativa* for Mutation Discovery and Crop Improvement. (**a**) Workflow for the TILLING population construction and the detection and confirmation of mutations in tridimensional pools. Created with BioRender.com. Tri-dimensional pooling diagram reproduced from Burkart-Waco et al. (2017) under Creative Commons license CC-BY-NC 2.5 (https://creativecommons.org/licenses/by-nc/2.5/). (**b**) Phenotypic characteristics of the TILLCANN population cultivated in permitted greenhouses (top); mutant seedlings displaying chlorosis (left middle), alterations in cotyledon number, and leaf phyllotaxis (right middle); and altered leaf morphology, and developmental abnormalities (bottom). (**c**) Confirmed EMS-derived mutations detected in C, D, and R amplicon pools and corresponding M2 families with the predicted effect on protein function. (**d**) Verification of mutations by Sanger sequencing in DNA of individual progeny of M2 mutant families where double peaks at shaded positions in a 1:1 ratio indicated the heterozygous state (presence of mutant allele) for genes *CsOLS1, CsTCP4,* and *CsMYB106* (left panels) and genotyping with PACE performed on 48 plants for the same genes with markers showing heterozygous individuals (red triangles) and homozygous (WT) individuals (blue triangles). (**e**) Phenotypic effects of the mutation of *CsTCP4* on phyllotaxis, leaflet number and leaf surface for mutant (left side panels) compared to wild-type (right side panels) plants

The EMS mutagenesis was conducted on F3 seed of a line derived from the cross of a CBD-accumulating genotype and the hemp cultivar Finola. Pilot studies were undertaken to determine mutagenesis conditions necessary to produce an adequate mutation frequency present in M1 plants (Fig. S1-S2). A large-scale mutagenesis was then performed using 2 h of pre-soaking combined with 3 h of EMS exposure at 150-, 200-, and 250-mM concentration. The M1 mutant plants derived from different concentrations (Table S1) were cultivated to obtain M2 seed in two cycles during which we observed developmental abnormalities, embryo lethality, and alterations in leaf morphology indicative of successful mutagenesis (Fig. 1b).

To assess our platform for efficiency in obtaining mutations, we first performed whole genome re-sequencing (WGRS) of six M2 plants (Table S2) and identified and annotated an average of 3,935 homozygous mutations per genome (Table S3; Fig. S3). However, mutations in exonic regions predicted to affect protein function (missense, splice site and stop-gained) were rare (mean of 134 per genome) occurring just 3.4% of the time. The ability to design amplicons in exons of target genes thus makes TbyS a highly effective approach to focus on these rare genic mutations. TbyS was conducted on a subset of 512 M2 families by designing 17 PCR amplicons derived from 13 target genes selected based on orthology and functional validation in other crop species (Table S4). Conserved genic regions in both genomes were selected for primer design to produce consistent amplification of PCR products from M2 DNA (Supplemental methods). The resulting PCR amplicons, ranging from 452 to 704 bp, were pooled tridemensionally to generate 24 DNA pools representing 64 families per pool) (Fig. 1a). Amplicons in pools were sequenced to a mean coverage of 11.3 × per M2 family that varied widely between pools (Table S5). Compared to mapping reads in a single reference genome, read mapping to simplified reference files from both cs10 and Finola genomes improved mapping efficiency (> 94%) (Tables S2, S5).

Using SNP calling procedures both with FreeBayes (Garrison and Marth, 2012) and a visualization approach (Supplemental methods) we detected 20 putative mutations in 22 M2 families. Sanger sequencing of amplicons in these families produced 17 clean trace files confirming 14 mutations in eight genes (Fig. 1c; Table S6) and yielding an 82% detection accuracy; similar to values obtained in platforms for other species such as tomato and barley (Gupta et al., 2017; Jiang et al., 2022). Based on 14 confirmed mutations and 4,474 Mbp of total sequence analyzed by TbyS, a mutation frequency of 1/320 kb was calculated; within the range of other diploid mutagenized populations created in tomato (1/237 – 1/737 kb) with a genome size (*ca.* 950 Mb) comparable to cannabis (Szurman-Zubrzycka et al., 2023).

To begin assessing mutant genotypes for a phenotype associated with variation in the target locus, we backcrossed the heterozygous M2 mutants and converted SNPs to allele-specific PCR markers for *CsOLS1*, *CsTCP4*, and *CsMYB106* to demonstrate their practicality for use in breeding programs (Table S7). All markers fit the 1:1 segregation ratio expected for BC_1_M2 plants where genotypes are either heterozygous or homozygous for WT alleles (Fig. 1d). Approximately half (*n* = 13) of the 25 BC_1_ plants in M2 family 200mM_99 associated with the mutation in TCP4-1 had marked phenotypic alterations; with tri-foliate, glabrous leaves showing reduced serration and venation compared to palmate, rugous, regularly serrated leaves typical of WT plants (*n* = 12) (Fig. 1e). The mutation is in a conserved sequence region close to a coiled secondary structure but appears not to provoke a change in protein folding (Fig. S4). Although the mechanism is unclear in our population, the TCP gene family is involved in the multilayer control of leaf development (Koyama, 2017) and complete linkage of the mutation in *CsTCP4* to altered leaflet number and morphology suggests an important role for this gene in the regulation of leaf development in cannabis as well. Functional mutations in highly conserved residues close to the enzyme active site were identified in *CsOLS1*, (Fig. 1c) which belongs to the type III polyketide synthases (PKS) family involved in the cannabinoid biosynthetic pathway (Taura et al., 2009). Targeted mutagenesis of other active site residues in OLS has been shown to produce alterations in olivetolic acid concentrations (Kearsey et al. 2020). The functional mutations in *CsOLS1* identified here are promising for their potential to either augment cannabinoid content for medicinal applications or diminish it for industrial hemp applications where exceeding limits of THC production are a concern.

The TILLCANN platform is a valuable new source for the creation of genetic diversity in cannabis. It provides a high probability of recovering mutations in genes of interest that will be useful for gene functional validation studies and in breeding programs through diversifying important traits related to both industrial and medicinal applications. Specifically, these efforts can fucus on optimizing cannabinoid profiles tailored for appropriate markets; understanding flowering time control as it is relevant for all cannabis market classes; improving disease resistance for pathogens of economic importance (e.g. powdery mildew, soil-borne pathogens, viruses, mycotoxins-associated pathogens); augmenting grain yield and quality, including oil and protein profiles, for the emerging cannabis food industry; and improving fiber quality traits with high heritability. As each mutant family contains an average of 3,935 background mutations, reduction of this load through backcrossing one to several generations may be necessary (Uauy et al., 2017) for breeding applications. Finally, large quantities of seed for most M2 families are available for performing forward genetic screens requiring many individuals for identifying mutants capable of germination and surviving in the presence of salt, drought or heavy metal stress. Improvement of abiotic traits fit squarely into the value proposition of industrial hemp as a sustainable and environmentally friendly crop.

## Supplementary Information

Supplementary Material 1.

Supplementary Material 2.

Supplementary Material 3.

## Data Availability

The main data supporting the results of this research are included in the supplementary material of this article. Sequencing data from the 24 amplicon-sequencing pools and the six WGRS libraries are available in the European Nucleotide Archive (ENA) repository under study ID PRJEB81779 [https://www.ebi.ac.uk/ena/browser/view/PRJEB81779]. Requests of specific mutant families or for the screening of mutations in additional genes can be extended to the corresponding authors.
