# Correction: TILLCANN: a TILLING platform in Cannabis sativa for mutation discovery and crop improvement

**DOI:** 10.1186/s43897-025-00221-8

**Published:** 2025-11-14

**Authors:** Diana Duarte‑Delgado, Konstantinos G. Alexiou, Marta Pujol, Cristobal Uauy, Nikolai M. Adamski, Victoria Vidal, Anthony Torres, Christopher Zalewski, Reginald Gaudino, Amparo Monfort, Jason Argyris

**Affiliations:** 1 Centre for Research in Agricultural Genomics (CRAG), CSIC-IRTA-UAB-UB, Campus UAB, Bellaterra, Barcelona, Spain; 2 IRTA (Institut de Recerca I Tecnologia Agroalimentaries), Barcelona, Spain; 3 John Innes Centre, Norwich Research Park, Norwich, NR4 7UH UK; 4 Front Range Biosciences, Lafayette, CO USA; 5 CIAT (International Center for Tropical Agriculture), Present address: Bean Program at Alliance of Bioversity International, Cali, Colombia; 6 Present address: Terpene Belt Farms, Oakland, CA USA; 7 Present Address: Cannabis Research Institute, Discovery Partners Institute, Chicago, IL USA

**Correction: Mol Horticulture 5, 54 (2025)**

**https://doi.org/10.1186/s43897-025-00176-w**

Following the publication of the original article (Duarte-Delgado et al. 2025), it is reported that Amparo Monfort should have been identified as corresponding author. The authorship has been corrected.

The original article (Duarte-Delgado et al. 2025) has been updated.
