# Supplementary material for: TILLCANN: a TILLING platform in Cannabis sativa for mutation discovery and crop improvement
Source: Mol Hortic. 2025 Oct 13;5:54. doi: 10.1186/s43897-025-00176-w (PMC12516893; doi:10.1186/s43897-025-00176-w)
Supplement: Supplementary file 1 — Supplementary Material 1. [file 43897_2025_176_MOESM1_ESM.docx]

**TILLCANN: A TILLING Platform in *Cannabis sativa* for Mutation Discovery and Crop Improvement**

Diana Duarte-Delgado^1†^ Konstantinos G. Alexiou^1,2^, Marta Pujol^1,2^, Cristobal Uauy^3^, Nikolai M. Adamski^3^, Victoria Vidal^1^, Anthony Torres^4††^, Christopher Zalewski^4^, Reginald Gaudino^4†††^, Amparo Monfort^1,2*^, Jason Argyris^1,2^*

^1^ Centre for Research in Agricultural Genomics (CRAG), CSIC-IRTA-UAB-UB, Campus UAB, Bellaterra, Barcelona, Spain.

^2^ IRTA (Institut de Recerca i Tecnologia Agroalimentàries), Barcelona, Spain

^3^ John Innes Centre, Norwich Research Park, Norwich NR4 7UH, United Kingdom

^4^ Front Range Biosciences, Lafayette, Colorado, USA

^†^ Present address: Bean Program at Alliance of Bioversity International and CIAT (International Center for Tropical Agriculture), Cali, Colombia

^††^ Present address: Terpene Belt Farms, Oakland, California, USA

^†††^ Present address: Cannabis Research Institute, Discovery Partners Institute, Chicago, Illinois, USA

^*^Corresponding authors: [jason.argyris@irta.cat](mailto:jason.argyris@irta.cat), [amparo.monfort@irta.cat](mailto:amparo.monfort@irta.cat)

**Supplementary Figure 1.** Germination (A) and seedling development (B) proportions for the three F3 lines assessed for large-scale mutagenesis experiments, and imbibition rate for TILL8 (C).


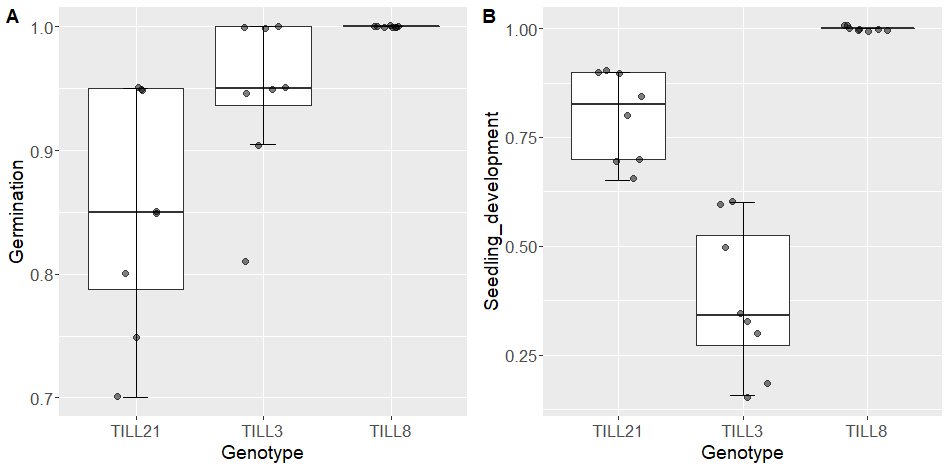

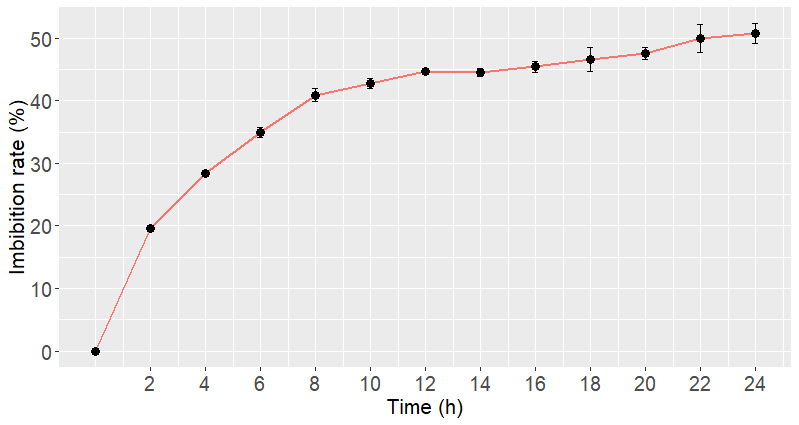


TILL8

**C**


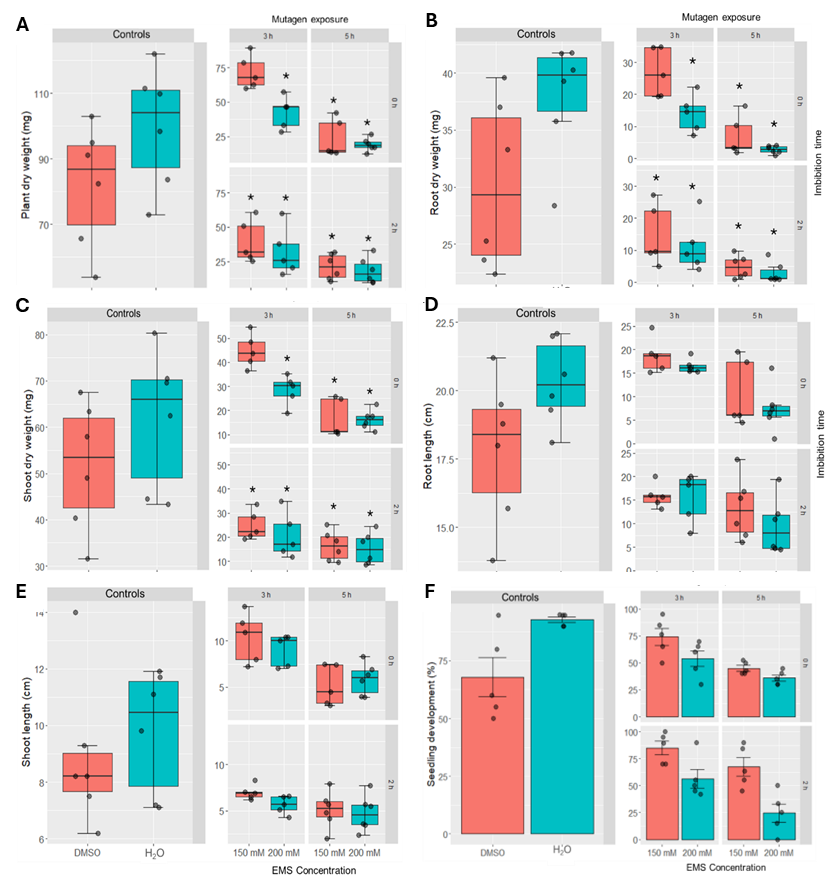


**Supplementary Figure 2.** Effects of two EMS concentrations combining two imbibition times on plant, root, and shoot dry weights (A, B, C) and root and shoot lengths (D, E) and seedling development (F) scored under controls H_2_O and DMSO (2%). Asterisks represent significant differences of the EMS treatments with the DMSO control (*p* < 0.05) in the growth-related parameters affected by mutagenesis conditions according to analysis of variance.



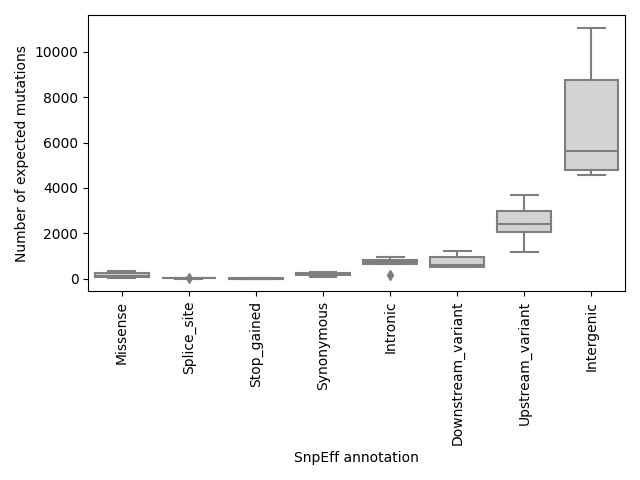


**Supplementary Figure 3**. SnpEff annotation (Cingolani et al., 2012) of canonical EMS-induced mutations detected by whole genome re-sequencing of six M2 plants.


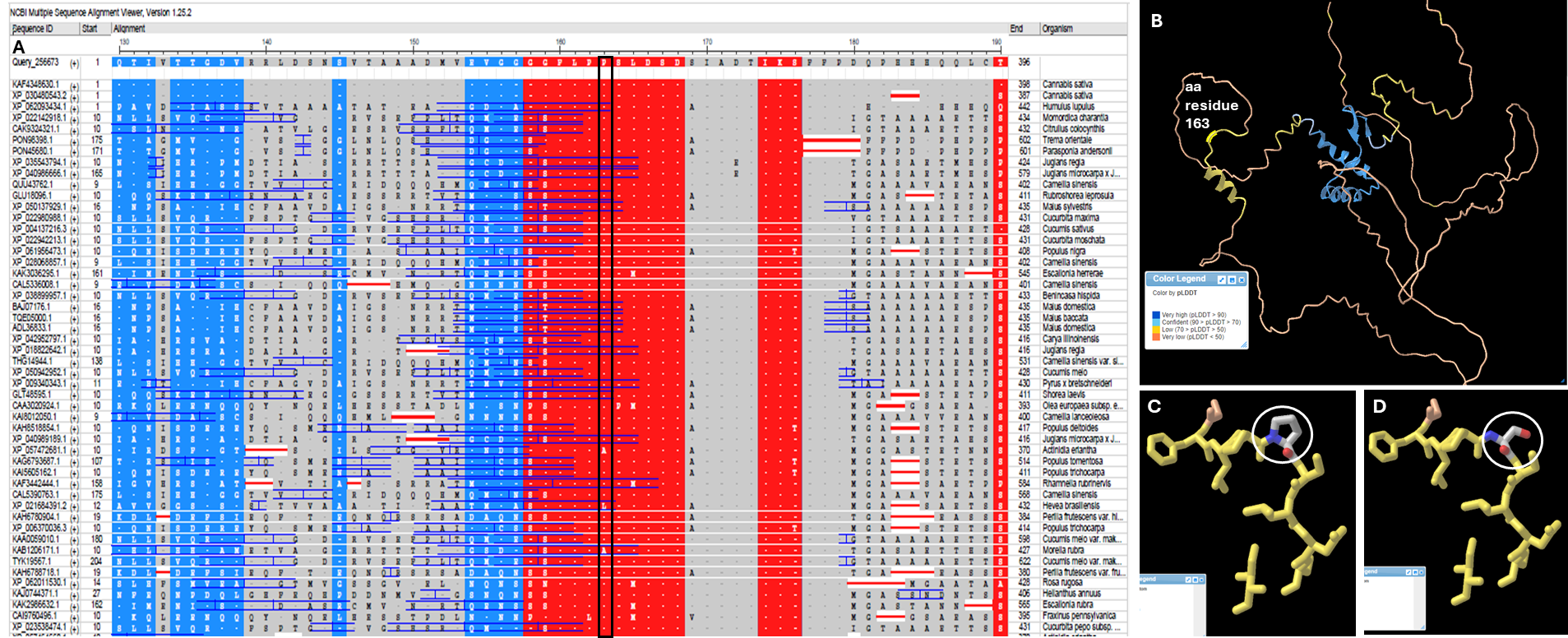


**Supplementary Figure 4**. Multiple alignment (A) using *CsTCP4* as anchor to compare sequence conservation from amino acid positions 130 – 190 across homologues in other species. Black box indicates the site of the mutation producing a P ->S substitution at position 163. Red indicates areas of high sequence conservation while blue and grey indicate less conserved regions. Protein structure as predicted by AlphaFold (Jumper et al., 2021; Varadi et al., 2024) with position 163 in yellow (B) and effects on folding structure of mutation resulting in a P ->S substitution at position 163 (circled) in WT (C) and mutant (D) proteins.

**References**

1. Cingolani P, Platts A, Wang L, Coon M, Nguyen T, Wang L, et al. A program for annotating and predicting the effects of single nucleotide polymorphisms, SnpEff: SNPs in the genome of *Drosophila melanogaster* strain w1118; iso-2; iso-3. Fly. 2012;6(2):80–92.
2. Jumper J, Evans R, Pritzel A, et al. Highly accurate protein structure prediction with AlphaFold. *Nature*. 2021;596(7873):583-589. doi:10.1038/s41586-021-03819-2
3. Varadi M, Bertoni D, Magana P, et al. AlphaFold Protein Structure Database in 2024: providing structure coverage for over 214 million protein sequences. *Nucleic Acids Res*. 2024;52(D1): D368-D375. doi:10.1093/nar/gkad1011
