# Supplementary material for: TILLCANN: a TILLING platform in Cannabis sativa for mutation discovery and crop improvement
Source: Mol Hortic. 2025 Oct 13;5:54. doi: 10.1186/s43897-025-00176-w (PMC12516893; doi:10.1186/s43897-025-00176-w)
Supplement: Supplementary file 3 — Supplementary Material 3. [file 43897_2025_176_MOESM3_ESM.docx]

**TILLCANN: A TILLING Platform in *Cannabis sativa* for Mutation Discovery and Crop Improvement**

Diana Duarte-Delgado^1†^ Konstantinos G. Alexiou^1,2^, Marta Pujol^1,2^, Cristobal Uauy^3^, Nikolai M. Adamski^3^, Victoria Vidal^1^, Anthony Torres^4††^, Christopher Zalewski^4^, Reginald Gaudino^4†††^, Amparo Monfort^1,2*^, Jason Argyris^1,2^*

^1^ Centre for Research in Agricultural Genomics (CRAG), CSIC-IRTA-UAB-UB, Campus UAB, Bellaterra, Barcelona, Spain.

^2^ IRTA (Institut de Recerca i Tecnologia Agroalimentàries), Barcelona, Spain

^3^ John Innes Centre, Norwich Research Park, Norwich NR4 7UH, United Kingdom

^4^ Front Range Biosciences, Lafayette, Colorado, USA

^†^ Present address: Bean Program at Alliance of Bioversity International and CIAT (International Center for Tropical Agriculture), Cali, Colombia

^††^ Present address: Terpene Belt Farms, Oakland, California, USA

^†††^ Present address: Cannabis Research Institute, Discovery Partners Institute, Chicago, Illinois, USA

^*^Corresponding authors: [jason.argyris@irta.cat](mailto:jason.argyris@irta.cat), [amparo.monfort@irta.cat](mailto:amparo.monfort@irta.cat)

**Material and Methods**

**Optimization of mutagenesis conditions**

The EMS mutagenesis was defined in F3 seed derived from the cross of an elite CBD-producing female and the hemp genotype Finola. The germination rate, seedling development and number of seeds from three F3 lines was assessed to select one line for massive mutagenesis. To define a duration for imbibition for the seeds, the imbibition rate was observed for 24 h and calculated as the percentage of water intake of seeds hourly as determined by an increase in seed weight after every hour of imbibition according to Unan et al. (2022).

After the imbibition rate study, a preliminary experiment was conducted to define the mutagenesis conditions. Two pre-imbibition times (0 and 2 h) and two mutagen exposures (3 and 5 h) were combined with two EMS concentrations (150 and 200 mM). EMS dilutions were prepared in 2% dimethyl sulfoxide (DMSO) and 0.3 mL/seed of solution was added to 100 seeds per treatment. Control treatments with distilled water and 2% DMSO were used. Seeds were surface sterilized with Captan 80% (2.5 g/L) fungicide for approximately one minute and then rinsed with tap water. Seeds were then imbibed in bottles with EMS solutions and placed on a rotary shaker at room temperature. The mutagenesis was inactivated by treating the seeds with sodium thiosulfate 100 mM in two rounds of 20 min in the rotary shaker. The seeds were then rinsed gently with tap water and distributed in Petri dishes (20 seeds/plate) with wet filter paper to assess seedling survival. Four days after mutagenesis, seedlings were transferred to trays with a substrate of peat moss, vermiculite and perlite in a 3:1:1 ratio in a growth chamber at 22 ºC and 18 h of light. A completely randomized design was established to sample five plants per treatment after 21 days to score height and dry weight of roots and shoots. A one-way analysis of variance was used to determine if the effect on growth-related traits from at least one EMS treatment was different from the others. The EMS treatments were compared against the DMSO control with pairwise comparisons considered significant after obtaining t-tests with Bonferroni adjusted *p-values* < 0.05. These analyses were carried out using RStudio v 2024.04.0 (RStudio Team, 2024).

For development of the TILLCANN population, 2 h of imbibition combined with 3 h of EMS exposure on 1,000 seeds each at 150, 200 and 250 mM was carried out. EMS was inactivated as described above. Following inactivation seeds were germinated in wetted rolled paper towels stored vertically in sealed plastic containers in a chamber at 24 ºC with 18 h of light. The mutagenesis was repeated twice to coincide with space limitations and desirable cultivation conditions in the greenhouse to produce M1 plants and M2 seed.

**Construction of the TILLING population**

At four days post-mutagenesis, seedlings were transferred to the greenhouse at CRAG and planted in 48-well trays with biodegradable pots filled with the substrate mix described previously. To maximize the probability of finding mutations, we prioritized transplanting seedlings from the 200- and 250-mM treatments. Males were visually discarded when floral primordia started to be conspicuous. Then, mutagenized females were transported to the IRTA research station in Caldes de Montbui (Spain; 41°37′54′′ N - 2°10′0.73″ W) and transplanted in 13 L plastic pots at 30 days post-mutagenesis. The pots were distributed in five elevated tables placed in a greenhouse under natural light conditions for the first experiment (harvested October 2022) and with supplementary light during the second experiment (harvested May 2023).

An M2 open-pollinated population was generated through the crossing of M1 females with wild-type males. Pollination was performed continuously for six weeks by shaking ten males across the plot with mutagenized flowering females. After ripening, the harvest of M2 families was performed by collecting the seeds from branches in individual trays using manual threshing. Seeds were deposited in brown kraft envelopes for drying at 23 ºC and 45% of relative humidity for two weeks then stored at 4 ºC under silica gel for long-term preservation.

**Mutation frequency assessed through WGRS**

The mutation frequency of the TILLING population was assessed at the whole-genome level through the WGRS analysis of six M2 plants derived from different families subjected to two EMS concentrations (i.e. 200mM_103, 200mM_219, 200mM_227, 250mM_57, 250mM_231, 250mM_247). DNA was extracted from 100 mg of young leaves grounded with liquid nitrogen using the DNeasy Plant Pro Kit (Qiagen, Hilden, Germany). After isolation, DNA was quantified using a Nanodrop 2000c spectrophotometer (Thermo Fisher Scientific, Waltham, MA, USA) and the integrity was visually assessed in 1.5% (w/v) agarose gels stained with ethidium bromide. Tubes with 1.5 to 2.1 µg of DNA were sent to BGI Genomics (Shenzhen, China) for library preparation and sequencing. Libraries with insert size of less than 800 bp were sequenced with a MGISEQ-2000 platform (MGI Tech, Shenzhen, China) to produce 30 Gbp of 100 bp paired-end reads for each one. Thus, each mutant was sequenced with an expected 34x coverage.

Raw re-sequencing data were aligned to cs10 reference genome assembly (Grassa et al., 2021) using bwa v0.7.17-r1188 (Li and Durbin, 2009). We used Picard v2.22.3 (Broad Institute, 2019) to mark PCR duplicates and add read groups in each alignment file. Variant calling was performed using GATK (v4.1.7.0) (Van der Auwera & O'Connor, 2020) and Deepvariant (Poplin et a., 2018; Yun et al., 2021) with default parameters, selecting reads with minimum mapping quality higher than 10. After obtaining the raw variants from both callers, we selected those GATK variants that were also detected by Deepvariant. This common set of variants was filtered with hard filtering criteria, separately for SNPs and INDELs, as suggested by GATK best practices (<https://gatk.broadinstitute.org/hc/en-us/articles/360035535932-Germline-short-variant-discovery-SNPs-Indels->) (DePristo et al., 2011) by using a minimum depth per-sample of 10, a minimum genotype quality of 20 and keeping sites with at least 90% of genotypes with data. To obtain the final list of putative EMS-derived mutations, a final set of three filters were applied to the variants. To detect a canonical EMS mutation in the WGS dataset we first selected positions with G<->A or C<->T transitions. Then we followed two strategies for getting the final list of putative canonical mutations. For the first strategy, we selected positions where both parents were heterozygous, five of the lines were heterozygous and one line was homozygous. This homozygous line would carry in theory the canonical mutation. For the latter strategy, we selected positions where both parents were homozygous, five of the lines were homozygous and the mutation-containing line was in heterozygosis. These variants were annotated with SnpEff (Cingolani et al., 2012).

**Detection of mutations by Illumina-based amplicon sequencing in a 3D-pooled TILLCANN population**

The workflow for the detection of chemical mutations is shown in Fig. 1. A total of 512 M2 families composed of 319 families from the 200 mM treatment, 176 from the 250 mM treatment, and 17 from the 150 mM treatment were included for DNA extraction and TbyS. Seeds were grown in groups of 64 families distributed in 8 columns and eight rows of biodegradable pots (11 x 11 cm) at elevated tables in the CRAG greenhouse (20 ºC mean temperature and 18 h light). Fifteen seeds per family were planted in the pots with the substrate described previously and covered with parafilm for 48 hours. Ten to 12 days after planting, one leaf disc of 6 mm diameter was collected from eight individual plants with a sharp hole punch. Then, eight discs from each family were homogenized with beads in a TissueLyser® disruptor (Qiagen, Hilden, Germany) to perform DNA extractions using the CTAB protocol (Doyle, 1991) with some modifications (Pereira et al., 2018). DNA quantification was performed with PicoGreen (Thermo Fisher Scientific, Waltham, MA, USA) using a Victor® Nivo™ (Perkin Elmer, Waltham, MA, USA) plate reader, followed by normalization to 20 ng/µl with a Mantis® liquid handler (Formulatrix, Bedford, MA, USA).

DNAs were pooled tri-dimensionally to construct 24 DNA pools (C1-C8, R1-R8, D1-D8) and diluted to 5 ng/µl for PCR reactions as described by Tsai et al. (2015). Pools were PCR-amplified using gene-specific primers. Owing to the hybrid nature of the genome of the TILLING population (i.e. a CBD-accumulating, cs10-like genotype and a fiber-use Finola genotype) conserved regions in both genomes were selected for primer design to produce consistent amplification of PCR products. Amplification conditions with KAPA HiFi HotStart Ready Mix (Roche, Basel, Switzerland) are described in the Supplementary Table S4 for each primer pair. To maintain adequate DNA concentrations for sequencing, PCR product concentrations were not normalized. Purified amplicons diluted in 52.5 µl of ultrapure water were quantified with PicoGreen and samples with concentrations greater than 0.3 ng/µl were used as template for Index PCR by merging for each sequencing pool 10 µl from each amplicon. The equal quantities of PCR products were combined in their respective pools, and combined amplicon pools were used for library preparation following the locus-specific primers protocol from the 16S metagenomic sequencing library preparation guide for the Miseq system (Illumina, 2013). Sequencing of libraries was performed on a MiSeq platform (Illumina, San Diego, CA, USA) that yielded 6.6 Gb of 2 x 300 bp paired-end reads.

Minimap2 (Li et al., 2018) was used to align the reads to a simplified reference file prepared with the sequence of the amplified genes retrieved from cs10 (Grassa et al., 2021) and Finola (Laverty et al., 2019) genomes. In each M2 family, a 3:1 ratio of WT:EMS-derived alleles are expected as 12 WT alleles were pooled with four mutated alleles in the tissue collection (i.e. four WT and four heterozygous plants with the mutated allele). Tri-dimensional pooling of DNA from the M2 families yields a pooling depth for mutation detection of 1:256 (four mutated alleles are expected in each pool with 512 families and 1024 alleles). Thus, the frequency change should rise above 0.0039 (4/1024) established as the detection limit to identify a mutation in an M2 family. We lowered this threshold to 0.0025 for the detection of variants using FreeBayes (Garrison & Marth, 2012) to account for scenarios of obtaining only three mutant alleles per pool, or in cases where mutant containing reads may have been discarded due to poor quality. The “pooled-continuous” option from the software was used to call variants simultaneously in the groups of libraries from each dimension. A mapping quality threshold of 20 was defined to consider reads for variant calling. After variant calling, a filter was applied to retain variants present in only one pool from each dimension. A constant of 2.8 was defined to multiply the alternative allele frequencies of the pools and distinguish putative chemical mutations from residual natural variability present as follows:

$${(AltC}_{i}>0.003) \mathrm{AND} \left( {AltC}_{i}{\geq2.8\times AltC}_{j} \right), for every j\neq i \epsilon\left\{ 1,2,\ldots,\left. 8 \right\} \right.$$

$for every i \epsilon\left\{ 1,2,\ldots,\left. 8 \right\} \right.$

where *AltC_i_* corresponds to the alternative allele frequencies from the *i*th C pool. Variants consistent in three or two dimensions were identified. An additional pipeline was used to score further putative EMS-derived mutations and to identify the missing pool for the variants observed in two dimensions. Read depth at each base was plotted across the amplicons from the different pools to analyze the consistency of sequencing quality across the pools and identify additional mutants through visualization based on alternative allele frequencies that exceeded threshold detection limits at corresponding positions. Graphs were generated based on the alternative allele frequency of C:T, G:A, T:C, and A:G changes across the amplicon length for each library. The *mpileup* tool from Samtools (Li et al., 2009) was utilized to score read bases with phred-base quality 20 at each position, followed by the calculation of the frequency of the alternative alleles at each site. Subsequently, the graph revealed mutations at positions where three libraries from different dimensions showed an increased frequency of mutated nucleotides.

**Confirmation of mutations in M2 families and individuals through Sanger sequencing**

The normalized DNA from the families diluted at 20 ng/µl was utilized in the PCR reactions for the confirmation of mutations through Sanger sequencing. Gene fragments were amplified using KAPA HiFi HotStart Ready Mix with the modification of some PCR conditions (Table S4). Sequencing was carried out at the Capillary Sequencing Facility from CRAG with an ABI 3730 DNA Analyzer (Applied Biosystems, Waltham, MA, USA) and fluorescent dye terminator detection. Examination of the sequence trace files was performed with Bioedit version 7.2.5 (Hall et al., 1999). These trace files were compared with the sequence of the unmutagenized genotype.

To reconfirm mutations in individual M2 plants, twenty-five seeds from unmutagenized line TILL8 and twenty-five seeds from each of the M2 families with functional mutations in *CsOLS1-1* and *CsOLS1-2* (200mM_114 and 250mM_201) as well as seeds of family M2 200mM_119 carrying a synonymous mutation in *CsOLS1-2*. We also germinated seeds of families 200mM_99, and 150 mM_29 for mutants *tcp4-1*, and *myb106*, respectively. Seeds were submerged in a prophylactic 80% thiram fungicide solution for approximately one minute and then rinsed with tap water. Seed were placed in petri dishes on saturated filter paper inside a translucent plastic box to maintain the humidity and incubated at 25 ℃ and 8 h of light. After three days, 16 developed seedlings for each family were transplanted and grown at 25 ℃ under 16/8 h diurnal cycle of light/darkness to maintain the plants in a vegetative state. DNA extractions, PCR, and Sanger sequencing were performed from young leaf tissue taken from each M2 plant as described previously to identify heterozygous female plants. After two weeks, lighting conditions were shifted to 12/12 h diurnal cycle to induce flowering. At flower initiation, heterozygous female mutant plants from each family were backcrossed with TILL8 male plants and BC1M2 seeds harvested after six weeks.

**SNP marker development**

To determine the zygosity of loci containing identified EMS-induced mutations, PCR Allele Competitive Extension (PACE®, 3CR Biosciences) markers were developed for each mutation in M2 plants as described previously (von Maydell, 2023) (Table S7). Primer Mix for each assay was prepared in final primer concentrations of 12 uM for A1, 12 uM for A2, and 30 uM for C1. DNA from M2 individuals with genotypes previously confirmed by Sanger sequencing were used to validate PACE® assays. Genotyping was performed using a LightCycler® 480 Instrument II (Roche), with detection type set to “Dual Color Hydrolysis Probe” and default PCR conditions. Results were analysed using LightCycler® 480 1.5.1.62 software. After validation, PACE® assays were conducted on BC1M2 DNA samples under the same conditions.

**Prediction of the effect of the EMS-derived mutations**

The prediction of the effect of the induced mutations was performed for those that were confirmed by Sanger sequencing. These mutations were identified in either introns or exons according to the structural annotation of the genes in cs10 and Finola. The codons with the variants were considered to identify amino acid changes or synonymous mutations. The PPVED software is a machine learning tool designed for plants used to predict the effect of the SAAS on the protein function (Gou et al., 2022). A prediction score (*Ps*) from 0 to 1 is produced for the substitutions to classify them as neutral (i.e. *Ps*<0.5) when an impact in the protein activity is not expected or functional (i.e. *Ps*≥0.5) when changes in protein function are presumed. To assess the extent of sequence conservation across species, 50 full-length *TCP4* homologues with the mutation were aligned using NCBI MSA Viewer 1.25.0. To visualize predicted protein structure of *CsTCP4*-1 and examine possible conformational changes induced by mutation, we used AlphaFold (Jumper et al., 2021).

**References**

1. Cingolani P, Platts A, Wang L, Coon M, Nguyen T, Wang L, et al. A program for annotating and predicting the effects of single nucleotide polymorphisms, SnpEff: SNPs in the genome of *Drosophila melanogaster* strain w1118; iso-2; iso-3. *Fly.* 2012;6(2):80–92.
2. DePristo M, Banks E, Poplin R, Garimella K, Maguire J, Hartl C, Philippakis A, del Angel G, Rivas MA, Hanna M, McKenna A, Fennell T, Kernytsky A, Sivachenko A, Cibulskis K, Gabriel S, Altshuler D, Daly M. (2011). A framework for variation discovery and genotyping using next-generation DNA sequencing data. *Nat Genet.* 43:491-498. DOI: 10.1038/ng.806.
3. Doyle J. DNA Protocols for Plants. In: Hewitt GM, Johnston AWB, Young JPW, editors. *Molecular Techniques in Taxonomy.* Berlin, Heidelberg: Springer; 1991. p 283–293.
4. Garrison E, Marth G. Haplotype-based variant detection from short-read sequencing. 2012. arXiv. 2012. doi:10.48550/ARXIV.1207.3907.
5. GATK Team. GATK best practices. In: *Genome Analysis Toolkit.* Broad Institute. 2024. <https://gatk.broadinstitute.org/hc/en-us/articles/360035535932-Germline-short-variant-discovery-SNPs-Indels>. Accessed Feb 2024.
6. Gou X, Feng X, Shi H, Guo T, Xie R, Liu Y et al. PPVED: A machine learning tool for predicting the effect of single amino acid substitution on protein function in plants. *Plant Biotechnol J.* 2022;20(7):1417–31.
7. Grassa CJ, Weiblen GD, Wenger JP, Dabney C, Poplawski SG, Motley S et al. A new Cannabis genome assembly associates elevated cannabidiol (CBD) with hemp introgressed into marijuana. *New Phytol.* 2021;230:1665–1679.
8. Hall TA. BioEdit: A user-friendly biological sequence alignment editor and analysis program for Windows 95/98/NT. *Nucleic Acids Symp Ser.* 1999;41:95-8.
9. Illumina. 16S metagenomic sequencing library preparation guide for the Miseq system. In: Illumina Support Center. 2013. <https://support.illumina.com/downloads/16s_metagenomic_sequencing_library_preparation.html>. Accessed Feb 2023.
10. Jumper J, Evans R, Pritzel A, et al. Highly accurate protein structure prediction with AlphaFold. *Nature.* 2021;596(7873):583-589. doi:10.1038/s41586-021-03819-2
11. Laverty KU, Stout JM, Sullivan MJ, Shah H, Gill N, Holbrook L, et al. A physical and genetic map of *Cannabis sativa* identifies extensive rearrangements at the THC/CBD acid synthase loci. *Genome Res.* 2019;29:146–56.
12. Li H, Durbin R. Fast and accurate short read alignment with Burrows–Wheeler transform. *Bioinformatics.* 2009;25(14):1754–60.
13. Li H, Handsaker B, Wysoker A, et al. The Sequence Alignment/Map format and SAMtools. *Bioinformatics.* 2009;25(16):2078–9.
14. Pereira L, Ruggieri V, Pérez S, Alexiou KG, Fernández M, Jahrmann T et al. QTL mapping of melon fruit quality traits using a high-density GBS-based genetic map. *BMC Plant Biol.* 2018;18:324.
15. Poplin R, Chang PC, Alexander D, et al. A universal SNP and small-indel variant caller using deep neural networks. *Nat Biotechnol.* 2018;36:983–7.
16. RStudio Team. RStudio: Integrated Development for R. 2024. In: <http://www.rstudio.com/>. Accessed 15 Jan 2024.
17. Tsai, H., Ngo, K., Lieberman, M., Missirian, V., & Comai, L. (2015). Tilling by sequencing. *Methods in molecular biology (Clifton, N.J.)*, 1284, 359–380. <https://doi.org/10.1007/978-1-4939-2444-8_18>
18. Unan R, Deligoz I, Al-Khatib K, Mennan H. Protocol for ethyl methanesulphonate (EMS) mutagenesis application in rice. *Open Res Eur.* 2022;1:19.
19. Van der Auwera GA, O'Connor BD. *Genomics in the Cloud: Using Docker, GATK, and WDL in Terra.* 1st ed. O'Reilly Media; 2020.
20. von Maydell D. PCR Allele Competitive Extension (PACE). *Methods Mol Biol.* 2023;2638:263-271.
21. Yun T, Li H, Chang PC, Lin MF, Carroll A, McLean CY. Accurate, scalable cohort variant calls using DeepVariant and GLnexus. *Bioinformatics.* 2021;36(24):5582–9.
